# Lung Deposition of Particulate Matter as a Source of Metal Exposure: A Threat to Humans and Animals

**DOI:** 10.3390/toxics13090788

**Published:** 2025-09-17

**Authors:** Joel Henrique Ellwanger, Marina Ziliotto, José Artur Bogo Chies

**Affiliations:** Laboratory of Immunobiology and Immunogenetics, Department of Genetics, Postgraduate Program in Genetics and Molecular Biology (PPGBM), Universidade Federal do Rio Grande do Sul (UFRGS), Porto Alegre 91501-970, RS, Brazil; marinaztto@gmail.com (M.Z.); jabchies@terra.com.br (J.A.B.C.)

**Keywords:** air, animal, anthracosis, ecopathology, metals, One Health, particulate matter, pneumoconiosis, pollution, potentially toxic elements

## Abstract

The deposition of particulate matter (PM) in the lungs is a health problem that primarily affects individuals working in mines and other highly polluted environments. People living in large cities also accumulate PM in their lungs throughout their lives due to the high levels of air pollution often observed in urban environments. In addition to the direct effects that the physical deposition of PM causes in the lungs, such as increased levels of inflammation and fibrosis, these pollutants can be associated with additional toxic effects, including genotoxicity and other molecular, cellular, and systemic alterations that can lead to the development of multiple diseases. This occurs because PM carries a number of toxic pollutants to the lungs, especially metals and metalloids such as arsenic, lead, cadmium, chromium, and mercury. Although the histopathological effects of occupational (pneumoconiosis) or environmental (anthracosis) deposition of PM in the human lungs are well described, little is discussed about how these morphological alterations can be a proxy for acute and chronic exposure to several toxic metals. Furthermore, the effects of PM–metal complexes on the health of animals, especially those living in urban environments, are often overlooked. In this context, this narrative review aims to discuss the impacts of lung-deposited PM–metal complexes on the health of multiple species, highlighting the broad effects caused by air pollution. Using the One Health approach, this article examines how environmental issues impact the health of both humans and animals.

## 1. Introduction

Particulate matter (PM) with a diameter of 10 μm or less (PM_10_), as well as those with a diameter of 2.5 μm or less (PM_2.5_), are among the main components of air pollution. The first particle type is considered coarse PM, while the second is considered fine PM. Particulate matter with a diameter of 0.1 μm or less (PM_0.1_) is classified as ultrafine particles, but they are not as frequent in the air as PM_2.5_ and PM_10_ [1]. These pollutants can enter the lungs through breathing. The smaller the PM, the greater the chance of these particles reaching deep parts of the lungs and then the bloodstream, thus being distributed throughout the body [1,2].

The lungs are unable to eliminate much of the inhaled PM, resulting in PM accumulation in terminal bronchioles and alveoli, which impairs breathing and facilitates the development of multiple lung-related pathological conditions, including chronic inflammation, fibrosis, and cancer. The accumulation of PM in the lungs is called “pneumoconiosis” when the condition’s etiology is derived from occupational exposure, with nomenclature variations depending on the type of pollutant to which the individual was exposed: coal workers’ pneumoconiosis (exposure to coal-related pollutants), asbestosis (asbestos), aluminosis (aluminum), chronic beryllium disease (beryllium), silicosis (silica), and hard metal pneumoconiosis (cobalt), among other variations [3,4,5,6,7]. The presence of combined pollutants can also be observed, for example, in the case of anthrasilicosis (anthracosis associated with silicosis) [8]. Indeed, in modern mines, miners breathe, in addition to coal dust, abundant mineralogical content and other types of pollutants [9].

People living in polluted urban environments also accumulate PM in their lungs over the years, a condition called “anthracosis” [5]. The term anthracosis is commonly used to designate the “non-pathogenic” deposition of PM (especially carbon) in the lungs, while pneumoconiosis refers to a group of lung diseases caused by the deposition of PM from varied occupational sources. Under clinical conditions, evaluating the life history of the affected individual is essential to differentiate the two conditions. In the literature, anthracosis can be treated in a broader way, especially when considering the deposition of PM in the lungs of humans and animals in an integrated manner. In summary, the term anthracosis is commonly, but not exclusively, used to refer to inhalation and deposition of coal-related pollution in the lungs. Therefore, it is important to stress that, in this review, we will use this term to refer to exposure to polluted environments in a broader sense.

Particulate matter accumulates especially, but not exclusively, in macrophages and in the interstitium around the terminal bronchioles, in the centrilobular region [5]. Notably, PM deposition in lung tissue is heterogeneous, which means that the particles are observed irregularly in different regions of the organ [10,11].

The lungs have mechanisms to remove inhaled PM, such as the action of macrophages and the mucociliary system, but these cellular and tissue strategies are not fully effective when the level of inhaled PM is very high or in cases of chronic exposure [11]. In people exposed to indoor or outdoor atmospheric PM during their lifetime, anthracosis can be observed microscopically or macroscopically, depending on its severity, as black pigments embedded in the lung tissue [5]. Anthracosis spots can be rounded or irregular in shape, with a diameter ranging from <1 mm to >50 mm [10].

The composition of PM varies depending on its emission source (e.g., vehicular traffic, industrial sources, biomass combustion, volcanic eruptions, dust clouds, building sites, tobacco smoke, and mining-derived coal dust) and the potential organic and inorganic elements that aggregate to PM after its emission. Particulate matter can carry a variety of microorganisms, polycyclic aromatic hydrocarbons (PAHs), and metals [1,12,13,14]. In other words, different types of PM (e.g., black carbon) act as “universal carriers” for multiple pollutants [12], and for this reason, these particles should be studied as a complex mixture of toxic pollutants [15].

Without considering the physical effects on PM deposition in the lungs, black (elemental) carbon as a constituent of fine PM may not be the main toxic agent of air pollution, but rather the other chemical compounds and metals that are found associated with PM [12]. Of note, fine particles (PM_2.5_), especially, show a high affinity with metals, including highly toxic elements such as lead [16]. It should be emphasized that ultrafine particles (e.g., PM_0.1_) also transport metals, are capable of reaching the deepest regions of the lungs, and exhibit a greater potential for translocation into the bloodstream, thereby reaching multiple organs [17,18,19,20]. Metals bind to PM during its emission or after its atmospheric release. The PM–metal complexes represent a major environmental and health problem worldwide [21].

**Figure 1:**
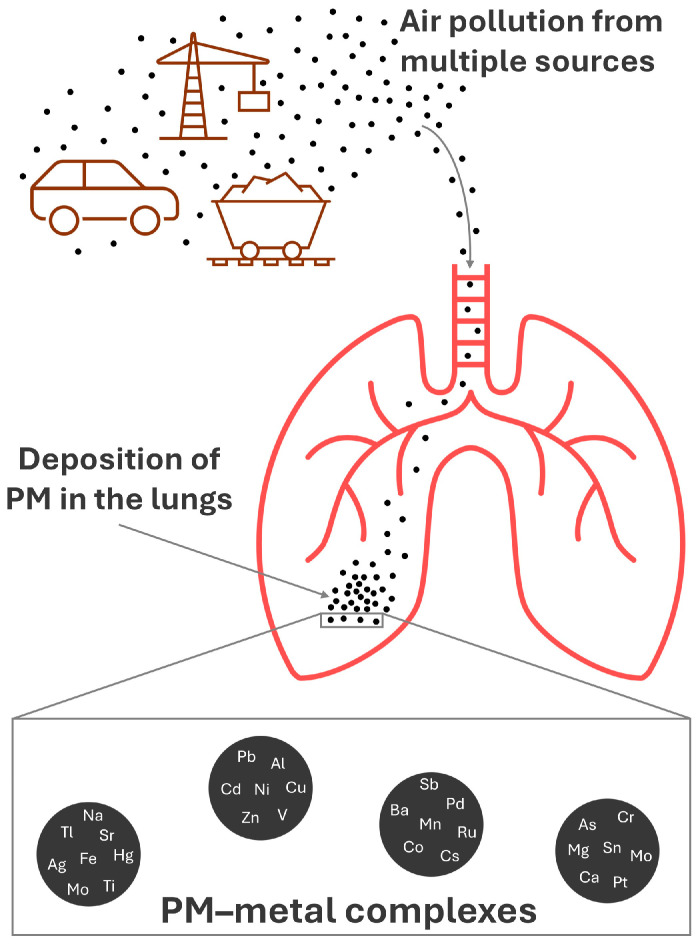
Pulmonary deposition of PM–metal complexes from different pollution sources. Ag: silver. Al: aluminum. As: arsenic. Ba: barium. Ca: calcium. Cd: cadmium. Co: cobalt. Cr: chromium. Cs: cesium. Cu: copper. Fe: iron. Hg: mercury. Mg: magnesium. Mn: manganese. Mo: molybdenum. Na: sodium. Ni: nickel. Pb: lead. Pd: palladium. PM: particulate matter. Pt: platinum. Ru: ruthenium. Sb: antimony. Sn: tin. Sr: strontium. Ti: titanium. Tl: thallium. V: vanadium. Zn: zinc.

A diversity of metals and semimetals is found bound to PM, including aluminum (Al), antimony (Sb), arsenic (As), barium (Ba), cadmium (Cd), calcium (Ca), cesium (Cs), chromium (Cr), cobalt (Co), copper (Cu), iron (Fe), lead (Pb), magnesium (Mg), manganese (Mn), mercury (Hg), molybdenum (Mo), nickel (Ni), palladium (Pd), platinum (Pt), potassium (K), ruthenium (Ru), silver (Ag), sodium (Na), strontium (Sr), tin (Sn), thallium (Tl), titanium (Ti), vanadium (V), and zinc (Zn) [1,2,15,21,22,23,24,25,26,27,28,29]. From here on, metals and semimetals will be referred to as “metals” for convenience. The levels of toxic metals found in PM often exceed the safe limits recommended by various global health and environmental protection agencies [21]. Moreover, even metals essential for the proper functioning of organisms, such as Fe and Zn, can become toxic in cases of high exposure. Therefore, deposition of PM in the lungs, whether in an environmental or occupational context, is a relevant source of exposure to several metals (Figure 1).

The quantity and types of metals present in PM will depend on the emission source and metals added to PM in the environment [1,28]. The metallic composition of PM–metal complexes also depends on the chemical characteristics of the emission source [29]. The presence of metals in PM is greater in areas where the sources of pollutants are linked to industrial activities, mining, fertilizer production, and intense vehicular traffic, among other metal-rich sources [1]. Differences in the profile of industrial activities in each region of the world significantly affect the proportions of metals present in PM. For example, countries in the Global South, where industrial activity is still growing, emit more Fe into the atmosphere than the Global North, where large-scale infrastructure development has already occurred [27]. It has been estimated that annually, fine PM from industrial activities alone releases up to 69,591 tons of metals into the atmosphere, with China, Australia, India, and Brazil being the countries that contribute most to these emissions [27]. In brief, anthropogenic actions are primarily responsible for the release of PM–metal complexes into the atmosphere [21]. Socio-environmental characteristics related to seasons of the year also affect the concentrations of metals found in PM, with a tendency towards higher metal concentrations in the winter compared to summer, although particularities exist in relation to specific metals and environmental contexts [16].

Although the toxic effects of metals and the problems caused by anthracosis and pneumoconiosis on human health are widely known, little is discussed about the role of pulmonary PM deposition as a source of acute and chronic metal exposure. Furthermore, there is little discussion about the effects of PM–metal complexes on the health of non-human animals, especially those living in urban environments and other places with high levels of air pollution. Considering these gaps in the literature, this article aims to review and discuss the impacts of PM–metal complexes, an environmental issue, on animal and human health, bringing metal toxicology closer to histopathology using a One Health perspective.

Since air pollution poses health risks to both humans and animals, the One Health approach, which integrates human, animal, and environmental health, is particularly well-suited to addressing this challenge. This approach emphasizes solving a broad environmental problem, in this case, air pollution, as an integrated way to benefit a variety of species [30,31,32,33].

## 2. Methodological Notes

This is a narrative review based on a non-exhaustive bibliographic search, with the intention of gathering bibliographical highlights on the topics covered in this article. The initial set of documents included in this review was selected from basic searches on PubMed [34] in July 2025, using the terms “animal”, “anthracosis”, “human”, “particulate matter”, “pneumoconiosis”, “pollution”, and “metal” in different combinations. Only articles published in English between 2010 and 2025 were considered eligible. Documents identified in the reference list of the articles selected in the initial search, as well as some documents available in the authors’ private libraries, were also considered eligible.

In the textual structure of this review, updated information on pneumoconiosis and anthracosis in humans is first presented. Data on anthracosis in animal populations is then discussed. The following chapters provide information on the toxic effects of PM–metal complexes, as well as potential solutions to this pollution problem. Finally, the article concludes with the main highlights of the reviewed content.

## 3. Anthracosis and Pneumoconiosis in Humans

Evidence of anthracosis and pneumoconiosis in humans is found in mummies from varied historical periods and world regions [5,35], showing that this is a health problem that has accompanied humans since ancient times, usually due to occupational exposure. In this sense, data covering the period between 1990 and 2019 indicate that coal workers’ pneumoconiosis continues to be a major global health issue, affecting millions of people around the world who work in polluted environments, with a reduction of only 3% of global incident cases in the period analyzed [36]. Pneumoconiosis associated with varied professional activities is reported in highly industrialized countries like the USA and some European nations (i.e., coal workers’ pneumoconiosis) [37,38], as well as in emerging economies, including China (i.e., welder’s and coal workers’ pneumoconiosis) [37,39] and Brazil [40], where agricultural practices and amethyst mining are important risk factors for the disease [40]. The types of metals to which individuals with pneumoconiosis are exposed depend on their specific occupational activities. For instance, welder’s pneumoconiosis is associated with exposure to metals such as Cd, Pb, Cr, and Zn [39], and Pb exposure can be a significant problem for coal workers [41]. More epidemiological data regarding pneumoconiosis from economically disadvantaged regions of the world, such as sub-Saharan Africa, are needed [42]. We add that the study of occupational risk factors for pneumoconiosis should consider other work activities, such as taxi drivers, who are highly exposed to pollution and show increased levels of metals, such as Hg, As, Pb, and Cd [43]. We speculate that chronic PM inhalation is one of the main sources of metals in these workers.

Beyond occupational contexts, chronic inhalation of PM is a problem that affects humans in their daily lives. Notably, indoor pollution increases the risk of anthracosis, as demonstrated in a study with individuals exposed to smoke from cooking activities in Iran, especially women [44]. Accordingly, a study by Lee et al. [45] showed an association between greater exposure to PM and increased blood levels of Cd and Pb in housewives from Ulsan, Korea. Increased PM inhalation due to physical activity also facilitates exposure to toxic elements, as demonstrated by the increased level of metals (i.e., As, Cd, Co, Cu, Mn, Ni, Pb, Se, Tl, Zn) on an average of 46.2% in the urine of athletes after exercise [46].

Analyzing images of the pleural surface from the upper and lower lobes of 413 autopsy cases from São Paulo (Brazil), Takano et al. [11] showed that the quantification of anthracosis in the lungs can be used as an indicator of lifetime exposure to air pollution, especially PM from traffic. Notably, São Paulo is a Latin American megacity with more than 12 million inhabitants, showing high levels of traffic-related air pollution. It is estimated that a person living in the city breathes in an amount of PM_2.5_ equivalent to smoking approximately five cigarettes a day [11]. Another study conducted with cadavers from São Paulo found an association between anthracosis and levels of alpha-emitting radon progeny polonium-210 among non-smokers, which suggests air pollution as a source of exposure to this highly toxic polonium isotope [47]. Furthermore, Attarchi et al. [48] reported a 32.1% anthracosis prevalence in 190 autopsy specimens from Guilan province (Iran), also describing a significantly higher prevalence of lung cancer, tuberculosis, and pulmonary histological changes in the group of anthracosis cases compared to controls. Combining histological techniques with synchrotron-radiation nano-X-ray fluorescence mapping, Falcones et al. [49] demonstrated that metals are physically deposited in areas with anthracosis in lung tissue, providing additional evidence that inhaled PM is a source of metal contamination and metal-related diseases.

Zhang et al. [50] evaluated the retention and leaching behaviors of several metals found in PM deposited in samples of human lung tissues of individuals nonoccupationally exposed to pollution in China. They showed that lung retention of metals is heterogeneous, varying depending on the metals: Al, Cd, Cr, Ba, Ni, Ti, Sn, V, and Sb (high retention); Pb, Mn, and Fe (moderate retention); Cu and Co (minor retention); Ca, Mg, and Zn (negligible retention). The moderate/low pulmonary retention of some metals, such as Fe, Ca, Mg, Zn, Cu, and Co, indicates that once PM is deposited in the lungs, a series of chemical, physical, and biochemical processes facilitates the leaching of these metals from pulmonary PM. Then, these metals can rapidly diffuse through lung tissue and potentially migrate to other parts of the body. Other metals can remain bound to the lungs for longer periods, being slowly released into the body, depending on the leaching behavior of each metal [50]. Therefore, the presence of PM in the lungs could be considered as a proxy of acute and chronic exposure to metals.

## 4. Anthracosis in Animals

Particulate matter is easily transported through the air, reaching aquatic and terrestrial ecosystems near and far from its emission source. Consequently, PM facilitates the exposure of numerous species to metals [1]. However, the knowledge about the impacts of pollution on animal health is still quite limited [51]. Considering that many animals live in urban environments with high levels of air pollution, it is very likely that they suffer from health problems associated with anthracosis in a similar way to humans, although these effects are usually overlooked.

The house sparrow (*Passer domesticus*) is highly adapted to urban environments [52]. Of 48 sparrows from Burdur (Turkey) analyzed by Ozmen et al. [52], 23 presented pulmonary anthracosis. Liu et al. [53] also observed cases of anthracosis in feral homing pigeons (*Columba livia*) in Chinese cities. Specifically, there was greater intensity of anthracosis in animals from Beijing compared to those from Chengdu, with a tendency for greater lung lesions found in pigeons from environments with higher atmospheric PAH concentrations. This body of evidence suggests that analysis of lung tissue of urban animals is useful for assessing the impacts of atmospheric quality on animals, in addition to being able to reflect, at least in part, human exposure. Of note, we speculate that analyzing anthracosis in animals may more realistically represent environmental pollution levels than analyzing it in humans, as animals do not have as many confounding factors as humans, especially smoking.

Animals that live closely with humans, such as dogs, are especially useful for the integrated study of the biological effects of pollution on the health of humans and animals. Recently, Oladipo et al. [54] investigated the prevalence of pulmonary anthracosis in 472 household dogs from Nigeria, in a study that covered the period between 2011 and 2020. They reported 150 cases of anthracosis (31.8%) among the dogs’ sample and suggested that analyzing lung tissue from household dogs is useful as a bioindicator of air quality for humans who share the environment with their dogs [54]. Thapa et al. [55] observed an anthracosis prevalence of approximately 50% (*n* = 25) in a sample of 52 stray dogs diagnosed with pulmonary distress from Kathmandu Valley, Nepal, a place with high levels of air pollution. Similar results were obtained by Mohamed et al. [56], who observed histological signs of anthracosis in 51.8% of 56 shelter dogs from Trinidad, Trinidad and Tobago. In dogs, air pollution-related anthracosis is associated with lung cancer [8]. In this sense, evaluating clinicopathological characteristics of primary lung cancer in dogs from Italy, Sabattini et al. [57] found an association between epidermal growth factor receptor (EGFR) expression and the presence of anthracosis, suggesting participation of EGFR signaling in the pathogenesis of air pollution-related lung cancer.

A 13-year-long investigation involving 350 zoo (*n* = 125), wild (*n* = 101), and companion animals (*n* = 124) of 61 different species showed that the presence of anthracosis is associated with the air quality of Jeollabuk-do Province, South Korea, a region with high atmospheric PM levels [58]. The frequency of anthracosis was highest in zoo animals (55.2%), followed by companion animals (45.2%) and wildlife (28.7%). This finding was associated with the fact that the zoo was located in an urban area [58]. Similarly, anthracosis was observed in 27 of 36 animals from Dhaka Zoo, Bangladesh, located in a polluted area [59]. A 3-year period study involving 115,186 slaughterhouse cattle from Arusha, Tanzania, reported a 7.3% frequency of anthracosis in 15,245 condemned lungs [60], showing that slaughterhouse animals are also subject to breathing poor quality air.

Studying tissues from accidentally killed animals is also a good source of information about anthracosis, without the need to intentionally sacrifice animals for research purposes. Nevárez-Garza et al. [61] reported mixed anthracosis in two road-killed wild coyotes (*Canis latrans*) living close to mineral extraction zones from San Luis Potosí, Mexico. The metals Fe and Cu were identified in animals’ lung tissue through histochemical techniques (Rhodanine and Perl’s Prussian blue stains for detection of Cu and Fe deposits, respectively) [61]. Anthracosis was observed in 12 of 34 (35%) lowland tapirs (*Tapirus terrestris*) killed by motor vehicle collision in the Cerrado biome, Brazil [62]. It is possible that automotive pollution and smoke from vegetation fires in the biome or from fire-dependent economic activities (e.g., charcoal production) were responsible for the anthracosis observed in these animals [62].

Using an ecopathology approach to evaluate the health conditions of 33 rodents (*Holochilus chacarius*) in rice agroecosystem from Pantanal wetland (Mato Grosso do Sul, Brazil), Rodrigues et al. [63] found anthracosis associated with inflammation in the lungs of 28 animals (84.8%), a result that influenced the rodents’ body condition. The anthracosis observed in these animals was likely caused by pollution emitted by smoke from anthropogenic fires in the wetland, which were facilitated by droughts that hit the Pantanal in recent years. This study also shows that even short-lived animals, such as small rodents, can accumulate significant amounts of PM in their lungs [63]. Deposition of PM in the lungs of small, short-lived animals also occurs in urban environments. For instance, Torres-Blas et al. [51] reported the presence of anthracosis in the lungs of American Eastern grey squirrels (*Sciurus carolinensis*) sampled in the London region, United Kingdom. Anthracosis is also observed in house sparrows (*Passer domesticus*) and pigeons (*Columba livia*), as discussed previously [52,53].

Although the number of studies concerning anthracosis in animal populations is limited, available information shows that PM deposition in animals’ lungs can affect body condition, inflammation levels [63], and is associated with lung cancer development [8]. Similar to what is observed in humans, we speculate that metals bound to PM are responsible, at least in part, for the animal health problems associated with PM exposure. Finally, although the use of animals as bioindicators of pollution levels is relevant, more studies are needed on the impacts of exposure to PM–metal complexes on animal health, as a purpose per se, helping to understand how pollution is affecting biodiversity.

## 5. Toxic Effects of PM-Bound Metals

**Figure 2:**
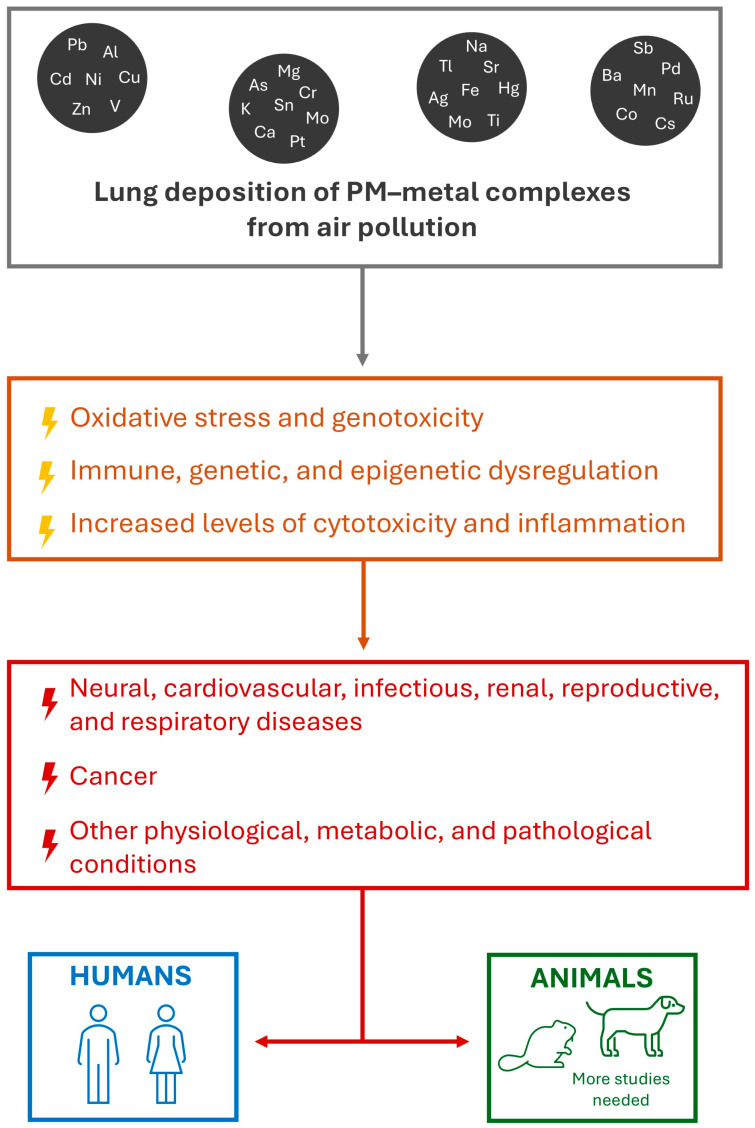
Biological impacts of air pollution-derived PM–metal complexes and their consequences for human and animal health. Ag: silver. Al: aluminum. As: arsenic. Ba: barium. Ca: calcium. Cd: cadmium. Co: cobalt. Cr: chromium. Cs: cesium. Cu: copper. Fe: iron. Hg: mercury. K: potassium. Mg: magnesium. Mn: manganese. Mo: molybdenum. Na: sodium. Ni: nickel. Pb: lead. Pd: palladium. PM: particulate matter. Pt: platinum. Ru: ruthenium. Sb: antimony. Sn: tin. Sr: strontium. Ti: titanium. Tl: thallium. V: vanadium. Zn: zinc.

The toxicity of PM derives from multiple associated pollutants, including PAHs and metals [14]. Prolonged exposure to metal-containing PM can cause severe and varied health problems, being more prominent in highly polluted areas where organisms are continually exposed to these pollutants [1]. Metal-containing PM may promote genotoxicity [64,65], immune, genetic, and epigenetic dysregulation [66,67], and cause increased levels of cytotoxicity, oxidative stress [14,68], and inflammation [66,69], which are processes associated with several diseases. A diverse body of evidence shows that exposure to PM–metal complexes is associated with neural, cardiovascular, and respiratory diseases, cancer, renal failure, reproductive abnormalities, among other pathological conditions [21,66,67,70,71,72,73,74,75]. Moreover, a set of studies from our group suggests that exposure to metal pollution can facilitate infections and the spread of pathogens among human and animal populations, as toxic metals such as Pb, Hg, and As harm the immune system and disrupt ecological networks that keep infectious diseases under control [76,77,78]. Figure 2 summarizes the effects of PM–metal complexes on human and animal health.

The cellular toxicity of PM is affected by particle size and its emission source, with PM_2.5_ from residential solid-fuel combustion, metallurgy industry, and transport-related sources being highly toxic. Brake-wear emissions during vehicle use and industrial processes are major sources of PM-related toxic metals [14]. Without considering other pollutants like PAHs, the toxic effects of metal-containing PM may result from the individual toxicity of the metals or the PM itself, or from the combined toxic effect of these pollutants. Some metals, such as Pb, are toxic at any concentration. Others, including essential metals (e.g., Fe, Zn), can become toxic when present in tissues in high concentrations. Stable oxidative forms of metals can bioaccumulate in tissues and interact with different biomolecules, forming toxic organometallic compounds [1].

Although the identification of total metals in PM is a traditional indicator of metal-related toxicity risks, it should be considered that the chemical species of each metal affects its bioaccessibility and bioavailability (e.g., solubility in lung fluid, capacity of absorption in the systemic circulation, ability to interact with biomolecules) and, consequently, its toxic effects [21,23,79]. For example, inorganic As can be retained in the lungs for long periods, producing chronic toxic effects [2]. Of note, a significant portion of metal species found in PM, such as As, Cd, Pb, and Zn, are soluble in water [23] and lung fluid [2]. Solubilized metals pose major health risks because they become bioavailable soon after being inhaled along with PM [23]. In this sense, the toxicity of metals should be assessed considering their bioavailability in lung fluid, which has specific chemical characteristics (e.g., presence of metal chelating agents) that affect the ability of metals to dissociate from PM and interact with biomolecules [80]. Furthermore, the synergistic and antagonistic effects between metals influence their toxic effects and should not be disregarded in metal-related risk assessments [79]. Finally, microbial activity in the pulmonary environment may also play a role that is still poorly understood in the release of metals from PM deposited in the lungs [79].

Using tools such as a transmission electron microscope and an X-ray photoelectron spectrometer equipped with a monochromatic Al-Kα excitation source, Yang et al. [27] showed an abundant and uniform distribution of several metals on the surface of fine PM. We speculate that these metals gradually dissociate from PM, depending on the physicochemical aspects of PM–metal bonds and the characteristics of the biological microenvironment (e.g., acidity) in which this complex is inserted. Therefore, we stress that PM deposition in the lungs is a potential source of both acute and chronic metal exposure.

In addition to the physiological and biochemical characteristics of organisms exposed to pollution, environmental factors also modulate exposure to PM-bound metals, impacting their biological effects. In urban environments, high temperatures increase the inhalation of PM–metal complexes, potentiating the associated health risks [81]. Extreme weather events induced by climate change will increase the remobilization and dissemination of metals in ecosystems, potentially increasing the exposure of humans and animals to the toxic effects of metal-containing pollutants [82,83]. This means that the toxic effects of metals will intensify as the spread of pollution is facilitated by climate change, providing further justification for intensifying control of the climate emergency as a way to protect the health of humans and animals in an integrated manner.

## 6. Potential Solutions

The evidence discussed in this article on the deleterious impacts of PM–metal complexes on humans and animals reinforces the need for air pollution control. Green areas, especially forest-based ecosystems, are effective in improving air quality in urban environments, where air quality must be constantly monitored by direct and indirect methods. Considering direct measurement, Brazilian authorities, for example, have already established limits for the amount of PM and Pb in air samples [84]. Complementary studies can help measure the amount of other toxic metals in the air. Considering indirect measurement, chemical and morphological analyses of plants can serve as bioindicators of the quantity and variety of metals present in the atmosphere [1,85].

The collaboration of industry in pollution control is essential and should involve reducing PM emissions through the development of less polluting production processes and the installation of efficient filters to retain pollutants [21]. Popular pressure on the productive sector to demand lower emissions of industrial pollutants is essential to guarantee environmental quality. However, although actions from individuals and organized communities are important for raising public awareness about pollution problems [21], improving air quality is a primary responsibility of public authorities and regulatory agencies in each country, through the development and implementation of policies that promote pollution control, in addition to encouraging less polluting economic development models [12]. Implementing economic models based on services and social well-being rather than the production of goods that require fossil fuels and metals is essential to reducing emissions of atmospheric and metallic pollutants, especially in countries of the Global North that have excessive consumption and production standards [86].

Not all individuals in the same population are exposed to the same load of pollutants. For instance, people living in peripheral areas of a large city like São Paulo spend more time commuting and are consequently exposed to a greater pollution load. In Brazil, as in other countries with high levels of social inequality, less socioeconomically privileged areas of cities may also show higher pollution levels due to various social issues, including environmental racism [87,88]. Therefore, reducing the time people spend commuting and consequently being exposed to traffic-related pollution is essential to controlling the harmful health impacts of pollution. This requires significant improvements in urban planning, including reducing social inequalities and implementing more effective, inclusive, and less polluting public transportation, as well as alternative mobility strategies [87]. Finally, including animal health parameters in pollution control strategies is crucial for pollution control to be beneficial for all living organisms.

## 7. Study Limitations

This study has some limitations. First, the article was not conducted as a systematic literature review, which means that some relevant studies may not have been included. Second, small-scale investigations in non-human animals, such as veterinary case reports, can provide valuable insights into anthracosis but are often found in the “gray literature”, which was not comprehensively covered here. Third, only a limited number of studies have simultaneously evaluated PM and metals in the lungs. Therefore, associations regarding the role of inhaled air pollutants as carriers of metals are drawn from a limited set of evidence and rely partly on indirect associations between lung deposition of PM and exposure to metals.

## 8. Conclusions

Particulate matter from various pollution sources contains several toxic metals. Inhalation of PM–metal complexes is one of the main routes of metal exposure, both in occupational and non-occupational environments. Therefore, both pneumoconiosis and anthracosis can be considered indicative of exposure to metal pollution. Once PM–metal complexes are deposited in the lungs, they release metallic elements that invade the bloodstream and are distributed to different organs, potentially causing various types of diseases and dysregulation at the molecular, tissue, and systemic levels. This problem affects humans and animals, but few studies evaluate the impacts of pulmonary deposition of PM–metal complexes on animal populations. More studies on this aspect are needed to better understand and mitigate the effects of pollution on the biodiversity crisis. Actions aimed at controlling atmospheric and metal pollution are necessary to protect human, animal, and environmental health.

## Data Availability

No new data were created or analyzed in this study.
